# FHB-Net: a severity level evaluation model for wheat *Fusarium* head blight based on image-level annotated aerial RGB images

**DOI:** 10.3389/fpls.2025.1549896

**Published:** 2025-09-15

**Authors:** Shuxin Zhu, Huayong Li, Shun Zou, Huanliang Xu, Zhaoyu Zhai

**Affiliations:** ^1^ College of Smart Agriculture (College of Artificial Intelligence), Nanjing Agricultural University, Nanjing, China; ^2^ Institute of Germplasm Resources and Biotechnology, Jiangsu Academy of Agricultural Sciences, Nanjing, China

**Keywords:** *Fusarium* head blight, severity evaluation, unmanned aerial vehicle, deep learning, attention mechanism

## Abstract

A leading concern for global wheat production, *Fusarium head blight* (FHB) can cause yield losses of up to 50% during severe epidemics. The cultivation of FHB-resistant wheat varieties is widely acknowledged as a highly effective and economical approach to disease management. The disease resistance breeding task depends on accurately evaluating the severity level of FHB. However, existing approaches may fail to distinguish among healthy and slightly infected wheats due to insufficient fine-grained feature learning, resulting in unreliable predictions. To tackle these challenges, this paper proposed the FHBNet model for evaluating the severity level of FHB under an end-to-end manner by simply using image-level annotated RGB images. In total, 6035 RGB aerial images taken from the wheat field were used to construct the dataset and each image was labelled by the light, moderate, or severe category. In FHBNet, we first utilized the multi-scale criss-cross attention (MSCCA) block to capture the global contextual relationships from each pixel, thereby modelling the spatial context of wheat ears. Furthermore, in order to accurately locate small lesions in wheat ears, we applied the bi-level routing attention (BRA) module, which suppressed the most irrelevant key-value pairs and only retained a small portion of interested regions. The experimental results demonstrated that FHBNet achieved an accuracy of 79.49% on the test se5t, surpassing the mainstream neural networks like MobileViT, MobileNet, EfficientNet, RepLkNet, ViT, and ConvNext. Moreover, visualization heatmaps revealed that FHBNet can accurately locate the FHB lesions under complex conditions, e.g., varying severity levels and illuminations. This study validated the feasibility of rapid and nondestructive FHB severity level evaluation with only image-level annotated aerial RGB images as an input, and the research result of this study can potentially accelerate the disease resistance breeding task by providing high-throughput and accurate phenotype analysis.

## 1 Introduction

Globally, wheat stands as a pivotal crop in both human and animal sustenance, contributing significantly to food security (Gruber, 2017). Its protein composition boasts ample amino acids to fulfill daily human dietary requirements (Chen, 2020). *Fusarium* Head Blight (FHB), primarily induced by *Fusarium graminearum*, poses a substantial threat to wheat, ranking among the most deleterious fungal ailments (Sari et al., 2020). The pathogen infiltrates the spikelet during flowering, causing premature desiccation and discoloration, thereby precipitating a notable decline in the wheat yield. Simultaneously, the toxins will also compromise the immune responses of both humans and animals (Femenias et al., 2020; Gilbert and Tekauz, 2000). Breeding the FHB-resistant cultivars stands as an effective strategy in combating this disease. However, the breeding process requires to evaluate the disease resistance of hundreds of wheat varieties. Exisitng methods of evaluating the disease severity level predominantly rely on visual observations by manually recording the proportion of infected wheat ears to the total ears in the field. This approach is labor-intensive and time-consuming. Consequently, there arises an urgent need for efficient approaches to assess the FHB severity levels.

In recent years, the analysis of plant phenotype has been driven by diverse neural network architectures (Mochida et al., 2019; Toda and Okura, 2019; Singh et al., 2018). Deep learning and computer vision have revolutionized the field of plant phenotyping by providing advanced tools for analyzing and interpreting various traits. These technologies have leveraged the power of artificial intelligence, which mimics humans to recognize patterns and make decisions. By learning from a sufficient amount of image data, neural networks can identify subtle phenotypic traits that are difficult for human observers to detect. This capability has proven particularly useful in precision agriculture, where timely and accurate assessment of plant characteristics can lead to better crop management and disease control strategies.

Various publications have reported the applicability of neural networks to wheat disease detection. Hamila et al. (2024) developed a 3D convolutional neural network to detect wheat FHB symptoms and accurately estimate the number of healthy and infected spikes. Roble et al. (2023) used classification algorithms to directly estimate the FHB scores without prior segmentation or detection of diseased wheat spikes. However, this study achieved a low accuracy of approximately 52% in discriminating the severity levels of FHB. Wang et al. (2023) developed a deep learning-based multi-model fusion system for real-time accurate diagnosis of wheat FHB, achieving high precision in wheat spike and lesion segmentation. The effectiveness of incorporating HSV color features as weighting factors in the wheat spike grading model was verified. Gao et al. (2022) collected images of individually isolated wheat ears and applied a pre-trained network for FHB prediction. Zhang et al. (2022a) developed a detection method for detecting the wheat FHB by integrating spectral and image features from raw unmanned aerial vehicle hyperspectral imaging data. Weng et al. (2021) proposed a method using HSI and deep learning networks to identify FHB severity levels by selecting specific wavelengths and applying a residual attention convolutional neural network, enabling high-precision classification of healthy and infected wheat kernels. Mi et al. (2020) developed C-DenseNet by utilizing the convolutional block attention mechanism (CBAM) and DenseBlock to extract traits of wheat stripe rust at varying severity levels. The attention heatmap confirmed that the proposed model was able to extract fine-grained features, thus accurately distinguishing disease levels with similar symptoms. Zhang et al. (2019) established a pulse coupled neural network to segment wheat ears infected with FHB. Nevertheless, this investigation solely addressed one spike within the image, rendering it impracticality for high-throughput detection in complex field conditions.

To boost the plant disease detection and classification accuracy, integrating the attention mechanism to deep learning models is one of the favorable strategies. Wei et al. (2025) introduced the dual branch channel attention and efficient spatial attention modules to ShuffleNetV2 to enhance feature learning of few-shot samples. The integration of the attention mechanisms boosted the classification accuracy by around 3%. Srinivasan et al. (2025) proposed DBA-ViNet for fruit disease detection and classification. Specifically, DBA-ViNet utilized a dual branch attention to capture global contextual information and fine-grained local lesion details simultaneously. Zang et al. (2025) developed a segmentation algorithm, RSE-Swin Unet, for wheat powdery mildew. The SENet attention mechanism was introduced to the baseline model Swin-UNet for capturing global and local features. The experimental result indicated that SENet was beneficial to identify irregular and small lesions. Bhujel et al. (2022) examined the effect of multiple attention mechanisms, including CBAM, Squeeze-and-Excitation (SE), Self-Attention (SA), and dual attention on the tomato leaf disease classification task. Recent works also reported more advanced attention mechanisms, such as high-frequency attention (Tian et al., 2025) context-guided attention (Yan and Li, 2025), multi-dimensional attention (Yang et al., 2025), cross-branch channel attention (Li et al., 2025), coordinate attention (Gu and Liu, 2025), etc. All these works confirmed the potential of integrating the attention mechanisms to deep learning models to achieve higher accuracy of detecting and classifying the plant disease.

Despite significant improvements in wheat FHB detection, most exisiting methods either collected images of isolated wheat spikes under constrained conditions or used algorithms to detect the percentage of spike and lesion pixels. However, these methods were not targeting to handling the real-world application scenarios and had weak generalization due to the scale differences of wheat spikes. Furthermore, the FHB severity evaluation task can be influenced by multiple factors. First, the quality of the data source is concerning. RGB images may be collected from different shooting angles under various illumination conditions. For instance, a healthy wheat spike taken under strong illumination usually has the similar visual characteristics as the one infected by FHB, both of which would appear color changes. Second, the FHB infected wheat spikes are usually distributed within the field plot unevenly and may be occluded by leaves and healthy wheat spikes. Therefore, aggregating the contextual information to enhance the feature learning is of great necessity. Although existing approaches have integrated attention mechanisms to tackle this issue, these attention mechanisms still experience weak generalization for FHB detection, particularly, may fail to adapt to the complex scenes in the wheat field. Third, at the early stage of FHB, the small spots on the spikes can be considered as small objects, which brings great challenges for the detection tasks.

To address the above-mentioned challenges in the FHB severity level evaluation task, we proposed an end-to-end deep learning model, namely FHBNet, which received an image-level annotated RGB image as an input and provided the severity level value of FHB as an output. The novel contributions of this study were summarized as follows.

- To enhance the contextual information of wheat ears, we developed a multi-scale criss-cross attention (MSCCA) block. It first replaced the standard 3×3 convolution kernels with ones that had a larger receptive field, enabling to effectively capture the long-term dependencies. Additionally, we introduced a multi-scale convolution module to adapt to variations in the orientation and scale distribution of wheat spikes, thus enhancing the spatial feature extraction. Concurrently, the MSCCA block performed a thorough semantic analysis of each pixel along vertical and horizontal directions, allowing each pixel to capture dependencies from its neighboring pixels, which greatly enhanced the pixel-level representational ability.
- As wheat ears were considered as small objects, we adopted the bi-level routing attention (BRA) block to further aid FHBNet to focus on the key regions within the feature map. Irrelevant key-value pairs were filtered out at a coarse region level. The BRA block was helpful to distinguish the FHB lesions among the complex background and locate the infected wheat ears more precisely.
- We conducted extensive comparative evaluations with several mainstream neural networks, including MobileViT, MobileNet, EfficientNet, RepLkNet, ViT, and ConvNext. The experimental results demonstrated the effectiveness of FHBNet in all evaluation indicators. Additionally, we validated the robustness of FHBNet under various settings (e.g., severity levels and illuminations). Through the heatmap visualization, it was evident that FHBNet can effectively address the detection challenges and achieve promising performance.

## 2 Materials and methods

### 2.1 Data acquisition and preprocess methods

**Figure 1:**
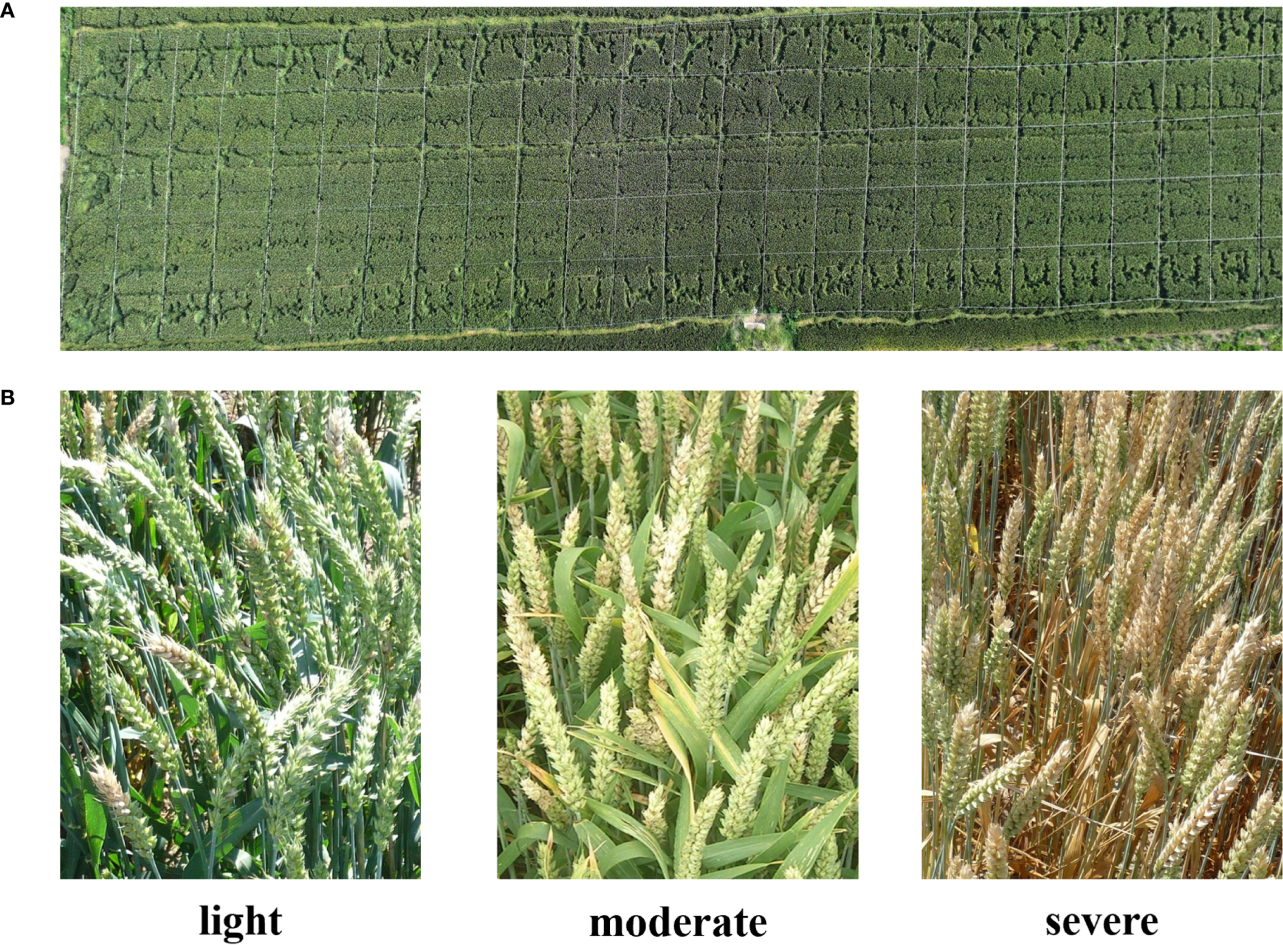
Data acquisition and labelling: **(A)** the plot setting. **(B)** example images of each category.

The experiment site was located in Huai’an, Jiangsu Province, China (119°01′20.74″E, 33°36′59.45″N). This region has a North subtropical humid monsoon climate with an average temperature at 14.5°C and an average rainfall at 960 mm. The wheat variety chose in this study was Huaimai 33 as the experiment material, provided by the College of Plant Protection, Nanjing Agricultural University. The experiment field was divided into 125 plots and each plot has 5.0 m both in length and width (see **Figure 1A**). All plots received the same agricultural management (e.g., irrigation, fertilization, weeding, etc.) except the disease control measure. FHB naturally occurred, rather than artificial inoculation. We applied different fungicide treatments to achieve each FHB disease severity level. For the control group, no disease control measure was adopted, resulting in the severe disease level. For the moderate and light disease levels, we applied the 200 g/hm^2^ and 400 g/hm^2^ of Phenamacril suspension concentrate, respectively.

The field data was acquired on 24 May, 2024, when the wheat was at the filling stage. We adopted a DJI Mavic 3 Pro unmanned aerial vehicle (UAV) for acquiring the RGB imagery. This UAV was equipped with a Hasselblad camera (4/3CMOS, effective pixels 20 MP), which fulfilled the requirement of data acquisition. The flight height of the UAV was set at 20 m and the flight speed was set at 1.5 m/s. After obtaining the raw video data, we manually filtered and cropped the video frames. To increase the size of the dataset, we also utilized an FHB dataset from the research contributed by Roble et al. (2023), resulting in a total of 6035 images. Each image was assigned by a category from light, moderate, and severe (see **Figure 1B**). **Table 1** listed the classification criteria and the number of samples included in all categories.

**Table 1:** The severity annotation scale and dataset distribution.

FHB severity	Infested spike area/%	The number of samples
Light	0-5	1754
Moderate	5-14	2012
Severe	14-100	2269

During training and validation, the images were resized to 224×224. To enhance the stability of the model and accelerate convergence, we normalized the data samples by adjusting features of different dimensions to similar ranges and pixel values to a specific interval. The dataset was divided to a training dataset and a test dataset by the ratio of 8:2.

### 2.2 FHBNet

**Figure 2:**
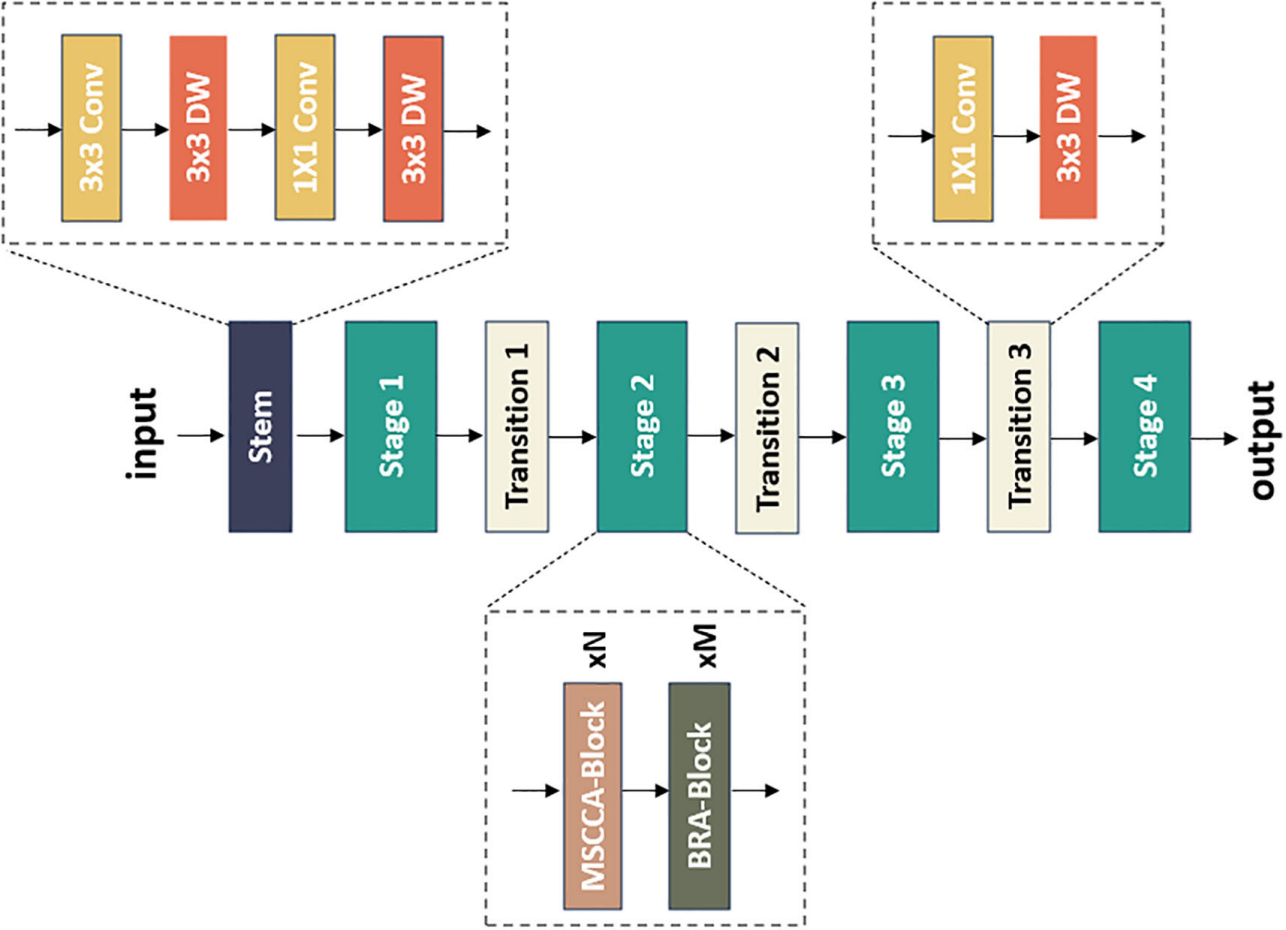
The architecture of the proposed FHBNet.

We proposed a novel model, namely FHBNet, that can accurately identify infected wheat spikes and determine the severity level of FHB. The overall architecture was depicted in **Figure 2**. The original image first entered the Stem layer, where a series of convolutional layers were stacked. After passing through the first 3×3 convolutional layer with a sampling rate of 2, we used a depth-wise separable 3×3 convolutional layer to capture the low-level features. Next, a 1×1 convolutional layer and a depth-wise separable 3×3 convolutional layer was employed to perform the downsampling operations. The image then progressed through the Stage layer, which was the core of FHBNet. It was responsible to locate the wheat spikes and extract the FHB features. The Stage layer included the MSCCA and BRA blocks, which will be elaborated in subsequent sections. The number of blocks in each stage was presented in **Supplementary Table S1**. It was worth mentioning that Stage 3 contained 18 MSCCA blocks, more than any other stage. This was due to the reason that in deep network architectures, deeper layers were often responsible for extracting more abstract features. Located in the deeper layers of FHBNet, Stage 3 dealt with more complex feature information. The MSCCA block, with its multi-scale and attention mechanism, effectively enhanced the model’s ability to abstract and represent features, enabling FHBNet to better understand and process the high-level semantic information. Last, the Transition layer served as a bridge between each stage, acting as an independent downsampling layer that doubled the channel dimensions and halved the image size.

### 2.3 MSCCA block

**Figure 3:**
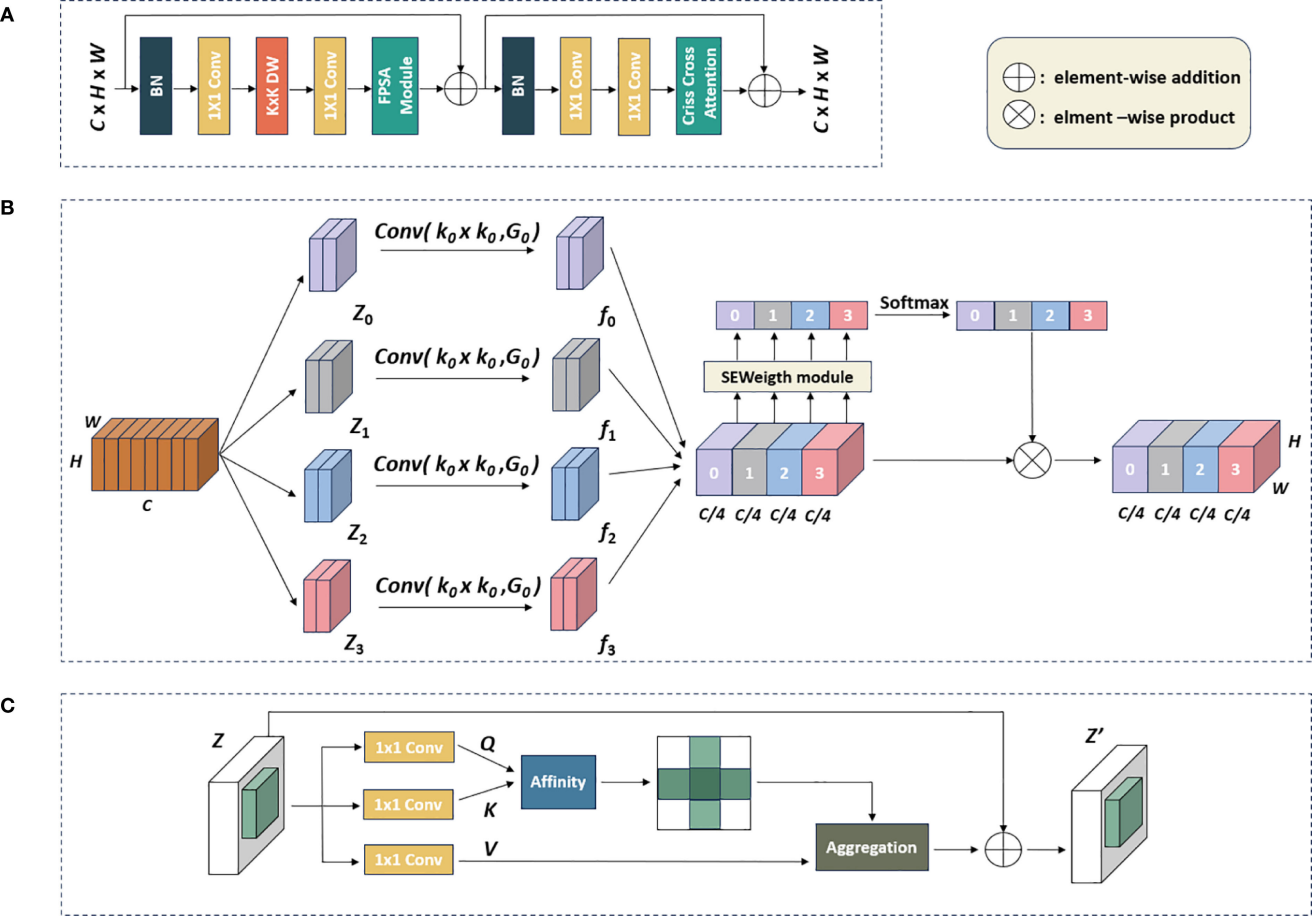
The workflow of the MSCCA block: **(A)** Structure of the MSCCA block; **(B)** the FPSA module; **(C)** the criss-cross attention module.

To better capture the information at various scales and model spatial contextual semantic relationships, we designed the MSCCA block and embedded it in all the Stage layers. The detailed design of the MSCCA block was shown in **Figure 3A**. In general, the MSCCA block consisted of several sub modules, mainly including the feature pyramid split attention (FPSA) and criss-cross attention (CCA) modules. The former was deployed to extract the multi-scale features, while the latter was helpful to enhance the extraction of the semantic information. Besides, we heavily employed 1×1 convolution operators in the MSCCA block to increase the model depth and introduce the non-linearity. Meanwhile, the 1×1 convolution operator can facilitate the cross-channel communication. In both FPSA and CCA modules, the residual connection was considered to tackle the network degradation issue (i.e., the gradient vanishing issue) (He et al., 2016). Additionally, we utilized large-kernel depth-wise separable convolutions to further broaden the receptive field. It was important to note that the size of the large-kernel convolutions varied in the MSCCA blocks across each stage. Specifically, the dimensions of the large kernels in Stages 1-4 were 31×31, 29×29, 27×27, and 13×13, respectively (Ding et al., 2022b).

Inspired by the design of EPSANet (Zhang et al., 2022b), we introduced the FPSA module in the MSCCA block, as shown in **Figure 3B**. Let *H*, *W*, and *C* denoted the height, width, and channels of the feature map, respectively. We first divided the feature map generated by the large kernel convolution into four branches along the channel dimension, represented by [*Z*_0_, *Z*_1_, *Z*_2_, *Z*_3_]. The convolutional kernels with different sizes were assigned to generate feature maps at various scales. To handle input tensors of different kernel sizes without increasing computational costs, the group convolution method was adopted. This means that the filters in the specific input channels only interact within these channels, greatly reducing the total number of trainable parameters. The multi-scale feature map was generated by Equation 1 as follows.


(1) $$f_{i}=\text{Conv}\left(Z_{i},\left(K_{i}\times K_{i},G_{i}\right)\right)$$

where Conv(·) denoted the group convolution operation. $Z_{i}$ denoted the $i^{th}$ feature map branch. $K_{i}$ denoted the size of the convolutional kernel in the $i^{th}$ branch, and $G_{i}$ denoted the group size in the $i^{th}$ branch. *K*_0_, *K*_1_, *K*_2_, and *K*_3_ are 3×3, 5×5, 7×7, and 9×9, respectively. *G*_0_, *G*_1_, *G*_2_, and *G*_3_ are 1, 4, 8, and 16, respectively. $f_{i}\;$ denoted the feature maps.

The feature maps generated from each branch were then concatenated along the channel dimension, formulated by Equation 2.


(2) $$\begin{array}{c} f=\text{Concat}\left(f_{0},f_{1},f_{2},f_{3}\right) \end{array}$$

where Concat denoted the concatenation of feature maps in a serial manner.

Subsequently, the attention weight vectors with four scales were obtained by extracting the channel attention weight information (Hu et al., 2018). This operation enabled the FPSA module to capture adequate contextual information in the feature map. In particular, each channel attention vector was concatenated and recalibrated through the Softmax function. The recalibrated weights and the corresponding feature maps were undergoing element-wise multiplication to produce a feature map with attention weights, enhancing its representational capability across multiple scales. The relevant formulas are defined by Equations 3, 4.


(3) $$\begin{array}{c} S_{i}=\text{SEWeight}\left(f_{i}\right) \end{array}$$

(4) $$F=f⊗\text{Softmax}\left(\text{Concat}\left(S_{0},S_{1},S_{2},S_{3}\right)\right)$$

where *S*_i_ denoted the attention weight vectors for each scale, ⊗ denoted element-wise multiplication. *F* denoted the feature map obtained after processing by the FPSA module.

After passing through the FPSA module, the feature map was then processed by the semantic feature enhancement module (i.e., the CCA module). Inspired by feedforward networks (FFNs) widely used in transformers (Kong et al., 2022; Liu et al., 2021) and MLPs (Ding et al., 2022a; Tolstikhin et al., 2021), we employed a CNN-style block composed of residual connections, batch normalization (BN), two 1×1 layers, with the addition of criss-cross attention at the end (Huang et al., 2019). Compared to the standard FFN, which used layer normalization before the fully-connected layers, BN had an advantage in that it can be fused into convolutions for efficient inference. BN normalizes the output of a layer for a given mini-batch by centering them around a mean of zero and scaling them to a standard deviation of one. BN effectively address the issue of internal covariate shift by ensuring that the inputs to each layer have a consistent distribution, regardless of the variations in the previous layer’s outputs.

The CCA module gathered the contextual information from each pixel along the vertical and horizontal paths. As shown in **Figure 3C**, the CCA module initially applied two convolutional layers with 1×1 filters on $Z\in R^{C\times H\times W}$ , producing two feature maps, *Q* and *K*, where $\left\{K,Q\right\}\in R^{C^{\prime }\times H\times W}$ and $C^{\prime }$ was the number of channels. At each position *u* in *Q*, a vector $Q_{u}\in R^{C^{\prime }}$ was obtained. Feature vectors from *K* corresponding to the same column or row at position *u* were also extracted, forming a set $\Omega_{u}\in R^{\left(H+W-1\right)\times C^{\prime }}$ . The attention map $A\in R^{\left(H+W-1\right)\times H\times W}$ was computed by applying a Softmax layer (that converted a vector of raw scores into a vector of probabilities) over the Affinity matrix (that quantified the similarity between two data points), which was calculated by Equation 5:


(5) $$d_{i,u}=Q_{u}\Omega_{i,u}^{\text{T}}$$

where, $d_{i,u}\in D$ represented the correlation between feature $Q_{u}$ and ${\text{Ω}}_{i,u}$ , where ${\text{Ω}}_{i,u}\in R^{C^{\prime }}$ was the $i^{th}$ element of $\Omega_{u}$ , and $i=\left[1,\ldots ,\left|\Omega_{u}\right|\right]$ , with $D\in R^{\left(H+W-1\right)\times H\times W}$ .

A third convolutional layer applied to *Z* generated $V\in R^{C\times H\times W}$ for feature adaptability. At each position *u* in *V*, a feature vector $V_{u}\in R^{C}$ and a set $\Phi_{u}\in R^{\left(H+W-1\right)\times H\times W}$ were obtained. The set $\Phi_{u}$ comprised feature vectors from *V* that were in the same column or row as position *u*.

The contextual information was aggregated by Equation 6:


(6) $$Z_{u}^{'}=\sum_{i=0}^{H+W-1}A_{i,u}\Phi_{i,u}+Z_{u}$$

where, $Z_{u}^{"}\;$ denoted the feature vector at position *u* in $Z^{\prime }\in R^{C\times H\times W}$ , $\Phi_{i,u}$ denoted the $i^{th}$ element of $\Phi_{u}$ , where $\begin{array}{c} i=\left[0,\ldots ,H+W-1\right] \end{array}$ , and $A_{i,u}$ denoted the scalar value at channel *i* and position *u* in the attention map *A*.

### 2.4 BRA block

Considering the complexity of backgrounds in the wheat field, and wheat spikes were small-scale targets, it was challenging to accurately locate the position of diseased wheat spikes. Additionally, the ability of most existing models to suppress background information was insufficient, which challenged the focus of the model on essential input features and increased its vulnerability to irrelevant background noise. To tackle this issue and enhance the model’s capability to detect small targets like wheat spikes and concentrate on the pathological features of FHB on these spikes, we incorporated a module, known as BiFormer, into the Stage section of FHBNet (Zhu et al., 2023). BiFormer is a recent computer vision algorithm integrated the attention mechanism from the Transformer architecture. It proposed a dynamic sparse attention mechanism by determining a small, relevant set of key pixels for each query pixel to attend to, rather than having every pixel attend to every other pixel.

**Figure 4:**
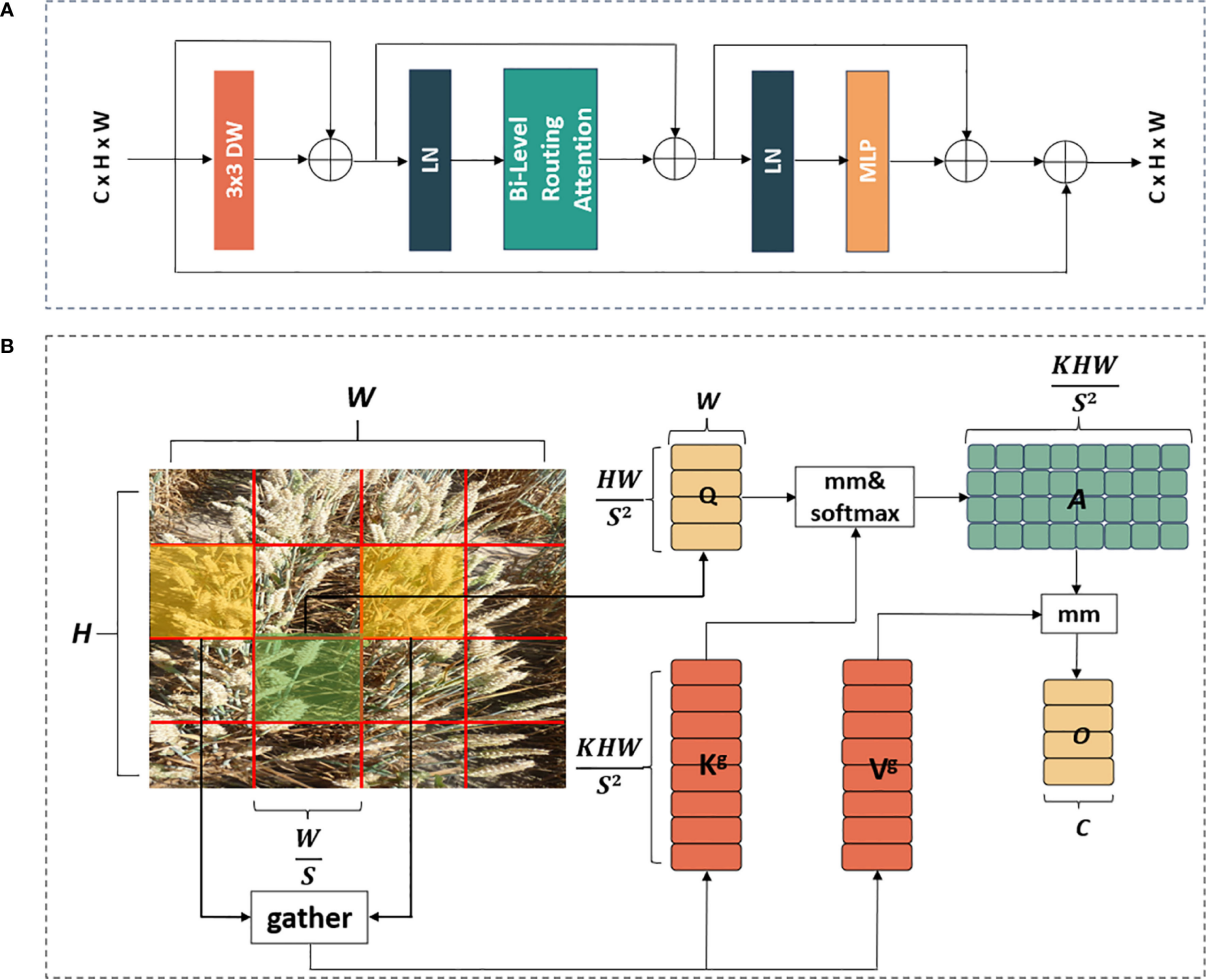
The workflow of BRA block: **(A)** overall structure. **(B)** bi-level routing attention module. In B, mm denoted the matrix multiplication operation.


The core of constructing the BRA block was the bi-level routing attention. As depicted in **Figure 4A**, the BRA block comprised a 3×3 depth-wise separable convolution, two layers of layer normalization (LN), a bi-level routing attention, three residual connections, and a two-layer MLP with an expansion ratio of 3. Here, the depth-wise convolution (denoted by DW in **Figure 4A**) was utilized to decrease the number of parameters and the computational complexity of FHBNet, while LN aided in accelerating the training process and enhancing the generalization of FHBNet. The MLP further processed and adjusted attention weights to intensify the focus of FHBNet on high-level features. Additionally, a residual connection between the original input and the final output was introduced, in addition to the original three residual connections, to further improve the model’s generalization capability. The computational process for the BRA block was outlined in (Equations 7–10).

(7) $${\hat{z}}^{l-1}=DW\left(z^{l-1}\right)+z^{l-1}$$

(8) $${\hat{z}}^{l}=BRA\left(LN\left({\hat{z}}^{l-1}\right)\right)+{\hat{z}}^{l-1}$$

(9) $$z^{l}=MLP\left(LN\left({\hat{z}}^{l}\right)\right)+{\hat{z}}^{l}$$

(10) $$z^{l}=z^{l}+{\hat{z}}^{0}$$

where ${\hat{z}}^{0}$ represented the initial input, ${\hat{z}}^{l-1}$ , ${\hat{z}}^{l}$ , and $z^{l}$ represented the outputs of the depth-wise convolution, the BRA module, and the MLP module, respectively.

The most critical component of the BRA block was the bi-level routing attention, a novel dynamic sparse attention mechanism. BRA first utilized query adaptivity to filter the input feature map, removing the least relevant key-value pairs in the coarse-grained areas, thus effectively identifying highly relevant key-value pairs for attention computation later. This not only greatly reduced the computational and storage resource consumption, but also enhanced the perception of the input content. The BRA block cares about only a small subset of relevant key-value pairs under a query adaptive manner, thus avoiding distraction from irrelevant ones.

As depicted in **Figure 4B**, the input feature map $X\in R^{H\times W\times C}$ was initially partitioned into S×S sub-regions, also known as patches, each containing *HW/S^2^* feature vectors. Subsequently, X underwent a change in shape to obtain $X^{r}\in R^{S^{2}\times \left(HW/S^{2}\right)\times C}$ . $X^{r}$ was then subjected to linear transformation to obtain three feature vectors Q , K , and V . The formulas for calculating *Q*, *K*, *V* can be expressed by Equations 11–13, respectively.


(11) $$Q=X^{r}W^{q}$$

(12) $$K=X^{r}W^{k}$$

(13) $$V=X^{r}W^{v}$$

Subsequently, a graph was constructed to obtain attention relationships between regions, determining the relevant regions. The specific implementation process was as follows. For each region, Q and V were processed by region averaging to obtain region-level $Q^{r}$ and $K^{r}\in R^{S^{2}\times C}$ . Then, the dot product of $Q^{r}$ and $K^{r}$ was computed to obtain the adjacency matrix $A^{r}\in R^{S^{2}\times S^{2}}$ , which measured the correlation between regions. The formula of calculating $A^{r}$ were expressed by Equation 14.


(14) $$A^{r}=Q^{r}{\left(K^{r}\right)}^{T},\;A^{r}\in R^{S^{2}\times S^{2}}$$

Next, at the coarse-grained level, the least relevant tokens in $A^{r}$ were pruned, retaining the top *k* most relevant regions in $A^{r}$ to obtain the routing index matrix $I^{r}\in N^{S^{2}\times k}$ . The formula of calculating $I^{r}$ were expressed by Equation 15.


(15) $$I^{r}=topkIndex\left(A^{r}\right)$$

Subsequently, token-to-token attention was used at a fine-grained level. For a query in region *i*, this attention focused only on the *k* routing regions indexed by $I^{r}\left(i,1\right),I^{r}\left(i,2\right),\ldots ,I^{r}\left(i,k\right)$ in the routing index matrix $I^{r}$ , and collected all the *K* and *V* tensors from these *k* regions to obtain $K^{g}$ and $V^{g}$ . The formula of calculating $\;K^{g}$ and $V^{g}$ were expressed by Equation 16.


(16) $$K^{g}=Gather\left(K,I^{r}\right),V^{g}=Gather\left(V,I^{r}\right)$$

Finally, attention processing was applied to the collected $K^{g}$ and $V^{g}$ , and a local context enhancement term *LCE (V)* was added to obtain the output tensor *O*. The formula of calculating *O* were expressed by Equation 17.


(17) $$O=Attention\left(Q,K^{g},V^{g}\right)+LCE\left(V\right)$$

The function *LCE*(·) was parameterized using a depth-wise convolution.

### 2.5 Experimental environment and training details

All experiments in this study were conducted under the same hardware and software configurations to ensure fairness. The experiments were performed on a server running Ubuntu 20.04, equipped with an Intel^®^ Core™ i7 central processing unit (CPU) and an NVIDIA GeForce RTX 3090 with a GPU memory size of 24 GB. Additionally, we utilized CUDA 11.3, cuDNN 8.9.0, and PyTorch 1.10.0 as the deep learning framework.

We utilized the Adam optimizer (Kingma and Ba, 2014) for training, with the momentum set at 0.9. Additionally, in 250 epochs, we set the batch size to 36 and employed a cosine annealing schedule (Loshchilov and Hutter, 2016) to adjust the learning rate, starting from $1\times 10^{-3}$ and gradually decaying to $1\times 10^{-6}$ . This scheduling method reduced the learning rate in a cosine curve fashion, effectively preventing the network from settling into local optima by periodically refreshing the learning rate, thus promoting better convergence and stability.

### 2.6 Evaluation metrics

In the experiment, we used four evaluation metrics: accuracy, precision, recall, and F1 score. The proportion of accurately classified instances, both positive and negative, out of the total number of instances was referred as Accuracy . It reflected the overall correctness of the classifier. Precision was defined as the proportion of accurately classified positive instances among all instances classified as positive. Recall , on the other hand, was the proportion of accurately identified positive instances among all true positive instances. The F1−score combined Precision and Recall into a single metric by taking the harmonic mean of these two metrics. These definitions were given by (Equations 18–21). Accuracy , Precision , Recall , and F1−score were denoted by Acc , P , R and F1 , respectively, in the following evaluation results.

In these Equations, True Positives (TP) and True Negatives (TN) referred to cases where the true labels and false labels matched the predicted results, respectively. False Positives (FP) and False Negatives (FN) referred to cases where false labels were incorrectly predicted as true labels and true labels were incorrectly predicted as false labels, respectively.


(18) $$Accuracy=\frac{TP+TN}{TP+TN+FP+FN}$$

(19) $$Recall=\frac{TP}{TP+FN}$$

(20) $$Precision=\frac{TP}{TP+FP}$$

(21) $$F1-score=\frac{Precision\times Recall}{Precision+Recall}$$

## 3 Results

### 3.1 Ablation experiment

We first conducted the ablation experiments on FHBNet to validate the effectiveness of the individual modules.

The results in **Table 2** demonstrated that each module had a positive impact on the prediction performance. The MSCCA block utilized large-kernel multi-scale convolutions and criss-cross attention to effectively adapt to changes in the orientation and scale of wheat ears in the field. It also thoroughly extracted the spatial contextual relationships, enhancing per-pixel representational capability, which increased accuracy by 4.06%. The attention mechanism in the BRA block focused on the critical information within the feature maps and effectively captured small, diseased wheat ears, raising accuracy by 1.35%. The improved model saw an overall accuracy increase of 5.42%, with all detection metrics showing substantial improvements. The FHBNet model achieved the optimal performance across all metrics, indicating that each component significantly contributed to the overall performance of the model.

**Table 2:** Ablation experiment of FHBNet.

Blocks			Metric		
MSCCA	BRA	Acc/%	R/%	F1/%	P/%
×	×	74.07	74.07	74.03	74.21
✓	×	78.13	78.13	78.62	78.81
×	✓	75.42	75.42	75.63	76.17
✓	✓	79.49	79.49	79.54	79.94

### 3.2 The feature extraction process at each stage of FHBNet

To more clearly demonstrate the feature extraction process of FHBNet for diseased wheat spikes, we utilized GradCAM (Selvaraju et al., 2017) to visualize the feature maps at different stages, thereby providing a clear display of FHBNet learning curves.

**Figure 5:**
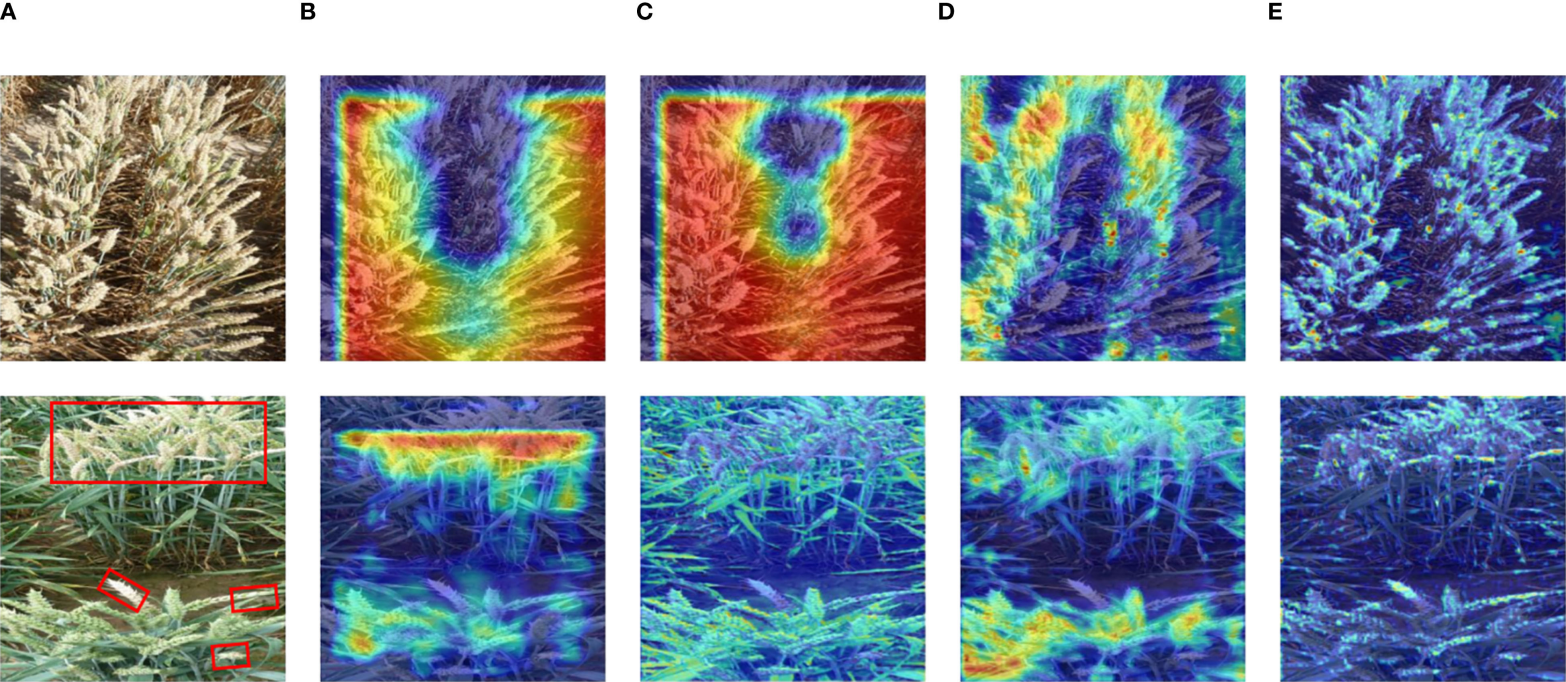
Heatmap visualization of FHBNet: **(A)** Original image. **(B)** Heatmap for Stage 1. **(C)** Heatmap for Stage 2. **(D)** Heatmap for Stage 3. **(E)** Heatmap for Stage 4. Note: the diseased wheat spikes are highlighted with red boxes in the second image.

As shown in **Figure 5**, we presented the test result using two example images. As the network deepened, FHBNet progressively extracted the features of wheat spikes, illustrating its effectiveness in feature extraction. This also highlighted the potential of FHBNet in detecting wheat spikes and identifying FHB. This formed the basis for the model to further discern pathological features on the spikes. According to the second test image, it was also evident that the model focused more on the diseased spikes.

### 3.3 Performance of FHBNet under different disturbances

**Figure 6:**
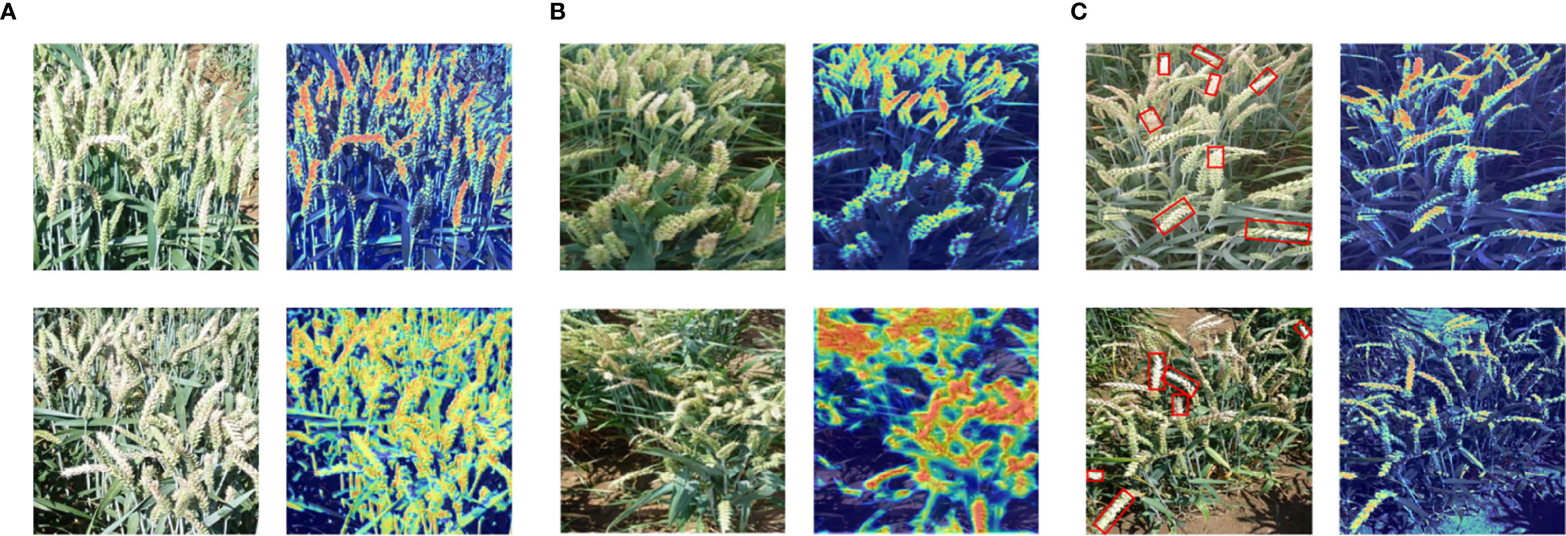
Heatmap visualization of FHBNet under different noise disturbances: **(A)** Strong natural light interference. **(B)** Different scales, with upper section indicated uneven distribution and the lower section denoted fallen wheat ears. **(C)** Intermingling of diseased wheat ears.

To verify the generalization ability of the FHBNet model, we visualized network feature maps under different noise disturbances. In natural conditions, overexposure of RGB images due to excessive sunlight is common. Therefore, we selected two examples of overexposed images for visualization. As shown in **Figure 6A**, even in overexposed conditions, the FHBNet model can still accurately locate infected wheat spikes. Meanwhile, when selecting resistant varieties, the shooting angles are often in non-ideal states, such as spikes concentrated at the edges or in the center, or spikes oriented towards various angles. Therefore, we selected two images: one with wheat samples concentrated at the edge and another with spikes fallen towards the photographer, corresponding respectively to the upper and lower parts of **Figure 6B**. The visualization results demonstrated that, under different angles and orientations of spikes, FHBNet can still precisely locate FHB lesions and effectively perform FHB detection. We observed that in cases of moderate infection, healthy, and diseased spikes often intermingle, which posed challenges for assessing the severity of FHB. To address this, we selected two images with a moderate severity of FHB to test whether our model can accurately locate FHB lesions under such disturbances. As shown in **Figure 6C**, examples on the left were the original images, with the diseased parts of the spikes framed in red. On the right were the visualizations, where the framed parts of the spikes were the FHB lesions, distinctly redder compared to the healthy spikes. The result demonstrated that the FHBNet model focused on the pathological features of FHB on the spikes and can accurately locate FHB lesions amid interspersed healthy and diseased spikes, thereby enabling it to assess the severity of FHB based on these findings.

### 3.4 Comparison with other methods

To further analyze the performance of FHBNet, we conducted comparative experiments on the same testing environment and dataset with several current mainstream and advanced neural network models, including ConvNext (Liu et al., 2022), ViT (Dosovitskiy et al., 2020), RepLKNet (Ding et al., 2022a), MobileNet (Howard et al., 2017), MobileViT (Mehta and Rastegari, 2022), and EfficientNet (Tan and Le, 2019). The basic information of compared models can be found in the note of **Supplementary Figure S1** in the supplementary file. To ensure a fair comparison, the hyperparameter configurations of these models were the same as FHBNet with Adam being the optimizer. The training Epoch was set to 250 and the batch size was 36. The cosine annealing schedule was adopted to promote better convergence and stability. **Table 3** displayed the test result, and we also plotted the loss and accuracy curves of the seven models for a clear comparison of accuracy (see **Supplementary Figure S1**).

**Table 3:** Comparison results of SOTA models.

Model	Light		Moderate		Sever		*F*1/%	*Acc*/%
	*P*/%	*R*/%	*P*/%	*R*/%	*P*/%	*R*/%		
**Ours**	**86.75**	**74.86**	**71.16**	**76.12**	**82.02**	**85.78**	**79.54**	**79.49**
MobileViT	82.84	80.01	64.25	70.65	81.37	71.65	75.63	75.42
MobileNet	82.39	82.86	65.37	66.67	78.40	76.61	75.12	75.08
EfficientNet	85.99	77.14	61.29	71.54	79.81	77.98	75.84	75.59
RepLkNet	82.43	69.71	64.25	66.17	77.41	84.86	74.03	74.07
ViT	72.41	60.12	46.57	64.18	74.42	58.72	61.69	60.94
ConvNext	84.62	50.29	45.66	78.61	76.39	50.46	60.44	59.93

Bold fonts indicated the best performance.

The results indicated that FHBNet achieved excellent performance, surpassing the second-best performing model by 3.7% in accuracy. We also observed that all models exhibited the lowest accuracy in recognizing moderate infection, as the complexity of the image background increased with healthy wheat and infected wheat intermingled, which interfered with the models’ discriminative capabilities.

**Figure 7:**
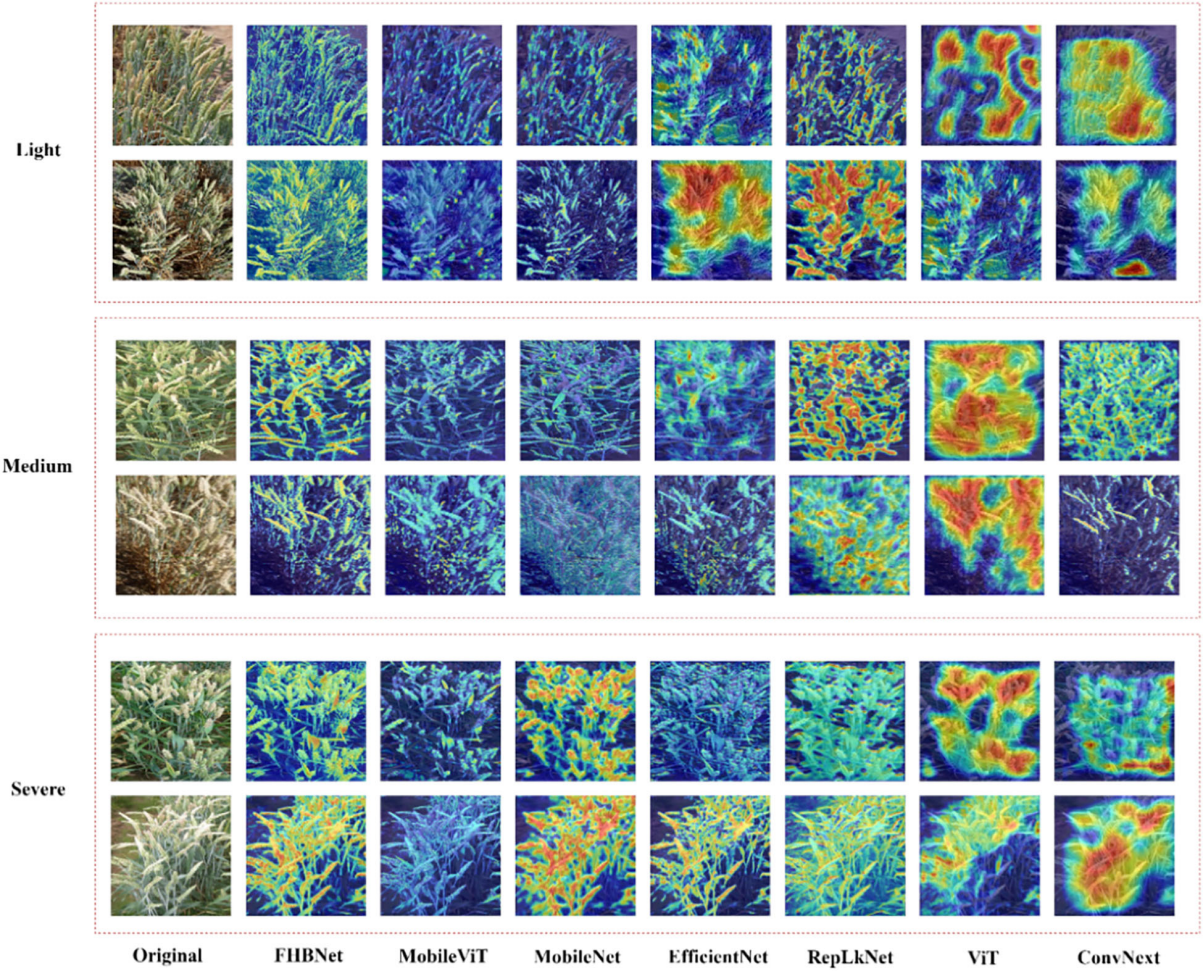
The heatmap visualization of all models.

Additionally, we selected two examples from each of the three FHB severity levels to visualize the heatmaps for all models. As shown in **Figure 7**, ConvNext and ViT performed the worst, only focusing on areas with color traits similar to FHB. RepLKNet, MobileNet, MobileViT, and EfficientNet showed better results, but still had instances of missed and false detections, and, in some cases, failed to detect the wheat spikes at all. In contrast, FHBNet was able to precisely locate the majority of the wheat spikes and further pinpoint the lesions.

## 4 Discussion

In this study, we developed and validated the FHBNet model for automatically assessing the severity of wheat FHB with image-level annotated RGB images as an input. We introduced the MSCCA block, which integrated the FPSA module and the CCA module to enhance the capability of FHBNet for feature extraction and semantic analysis. The multi-scale convolution module adapted to variations in the orientation and scale distribution of field wheat spikes, thus improving the efficiency of spatial feature extraction. We enhanced the model’s pixel-level representation by capturing the relationships between adjacent pixels. Subsequently, we introduced the BRA block to filter noise region from the feature map, achieving precise localization of small lesions on the spikes. In the experiments, FHBNet demonstrated superior performance, with accuracy and F1 score reaching 79.49% and 79.54%, respectively. FHBNet outperformed other advanced neural network models. Moreover, it maintained high recognition accuracy under various disturbances. These results indicated that FHBNet possessed strong generalization capability and robustness, enabling it to work stably under diverse field conditions.

We further analyzed models with the help of **Table 3**, **Figure 7**. The self-attention mechanism was a core advantage of ViT, enabling the model to capture relationships between different parts of the input image, regardless of their spatial positions in the image. This mechanism worked by computing the correlation between each image patch and all other patches, allowing ViT to understand the image globally rather than just locally. This was beneficial for the model to understand wheat images in complex backgrounds. However, by observing the result of ViT in **Figure 7**, we found that the heatmaps generated by ViT might have been too scattered, not clearly focusing on the details or pathological changes of wheat ears. The reason might have been that although the self-attention mechanism emphasized the global context, it might have overlooked the fine processing of extracting local features. When processing images of wheat ears, ViT might have overly focused on broad regions in the overall image rather than precisely focusing on subtle pathological changes on the ears. Additionally, compared to convolutional layers, self-attention lacked structured patterns of local receptive fields, which might have led to poor performance in extracting detailed features with local correlations.

The effect of MobileViT was slightly better than ViT. MobileViT combined the characteristics of ViT and convolutional networks, aiming to balance the global perceptual ability of Transformers with the efficient computation and local feature extraction ability of CNNs. This combination to some extent mitigated the drawback of ViT focusing only on global features. However, by observing the result of MobileViT in **Figure 7**, we found that some heatmaps displayed overly smooth activation regions, which might have reflected a ‘blurring’ phenomenon in the model’s feature recognition process, meaning that the model failed to distinguish between different structures and boundaries in the image. Specifically, the global attention of the Transformer layers might have to some extent suppressed the local sensitivity of the convolutional layers, leading to visually smoother and blurrier feature representations. This was detrimental to the model’s focus on wheat ears and their pathological features.

RepLKNet was a convolutional neural network that used large kernel convolutions to expand the model’s receptive field and improve its ability to capture image features. This structure could better capture long-range dependencies in images, establish a good semantic space, and thus be able to locate wheat ears in complex backgrounds. Although the RepLKNet model successfully located the overall position of wheat to some extent, the range and contours of its activation areas were obviously larger than the actual wheat area in some samples. This might have been because RepLKNet used large kernel convolutions, which had a relatively wide receptive field. While this wide receptive field helped capture global semantic information in images, it might have also led to a decrease in accuracy in detail localization, especially in tasks that required precise delineation of small or fine features, which was detrimental in the severity discrimination task of FHB.

MobileNet achieved high recognition accuracy. However, as shown in the MobileNet column in **Figure 7**, it performed particularly poorly in the moderate class. The reason might have been that in the two examples of the moderate class, the spatial distribution of wheat was complex, and MobileNet was designed to be lightweight and efficient, it might not have been able to capture certain details and semantic information in complex images, especially for tasks that required understanding global context and were easily interfered by irrelevant background information. For example, MobileNet focused on the leaves, which was due to the model’s failure to capture semantic information in the image, being interfered by irrelevant background information. Through the comparison in **Figure 7**, FHBNet could more completely and accurately extract wheat ears compared to other state-of-the-art models, which was important for further locating lesions on wheat ears and identifying the severity of FHB.

Additionally, our core advantage lied in the model’s ability to directly learn features related to the severity of FHB from raw RGB images, without relying on annotated pixel-level annotations. This significantly reduced the cost of data labelling, while also enhancing the model’s feasibility for practical applications (Kumar and Kukreja, 2022). Furthermore, the end-to-end nature of the model allowed for easy integration into existing agricultural monitoring systems, facilitating real-time disease prediction.

Despite the significant achievements of FHBNet in this study, there were still areas that warranted further exploration and improvement. First, under certain disturbances, there were still instances of missed detection of infected wheat spikes. As shown in **Supplementary Figure S2**, awns and weeds extensively cover the diseased spikes, and their color resembled that of the disease traits, making them difficult to distinguish. This complicated FHBNet to accurately assess the severity of FHB. In order to further enhance the prediction performance and robustness of FHBNet, we would further enhance the ability of extracting fine-grained features. For instance, to reduce the background interference on predictions, one could try the multi-granularity feature fusion approach (Xu et al., 2024; Su et al., 2024). Alternative approaches to enhance feature learning can also refer to knowledge distillation (Liu et al., 2024; Zhang et al., 2024). Second, FHBNet had a higher computational load compared to classic lightweight models and required more computing resources. Potential future directions for this research involve minimizing the model size through model compression and model pruning techniques to enable integration into edge devices (Yan et al., 2024; He et al., 2022). Such integration could aid in the onsite selection of FHB resistant varieties, thereby improving breeding efficiency. Last, optimizing the model for embedded devices could broaden its application in precision agriculture scenarios.

## 5 Conclusion

The model proposed in this study has achieved notable success in extracting FHB resistance phenotypes, providing a reliable basis for screening wheat resistant varieties. This research outcome is not only of theoretical importance but also provides practical decision support for wheat breeding, accelerating disease-resistant breeding efforts, and potentially providing new breakthrough directions for agricultural production.

## Data Availability

The raw data supporting the conclusions of this article will be made available by the authors, without undue reservation.
