# Supplementary material for: FHB-Net: a severity level evaluation model for wheat Fusarium head blight based on image-level annotated aerial RGB images
Source: Front Plant Sci. 2025 Sep 15;16:1549896. doi: 10.3389/fpls.2025.1549896 (PMC12477448; doi:10.3389/fpls.2025.1549896)
Supplement: Supplementary file 1 [file Supplementaryfile1.docx]

Supplementary Material

**Table S1**. The quantity of corresponding modules in each Stage.

| Stage | Block numbers | | Output size |
| --- | --- | --- | --- |
|  | MSCCA | BRA |  |
| 1 | 2 | 2 | 128×56×56 |
| 2 | 2 | 2 | 256×28×28 |
| 3 | 18 | 4 | 512×14×14 |
| 4 | 2 | 2 | 1024×7×7 |


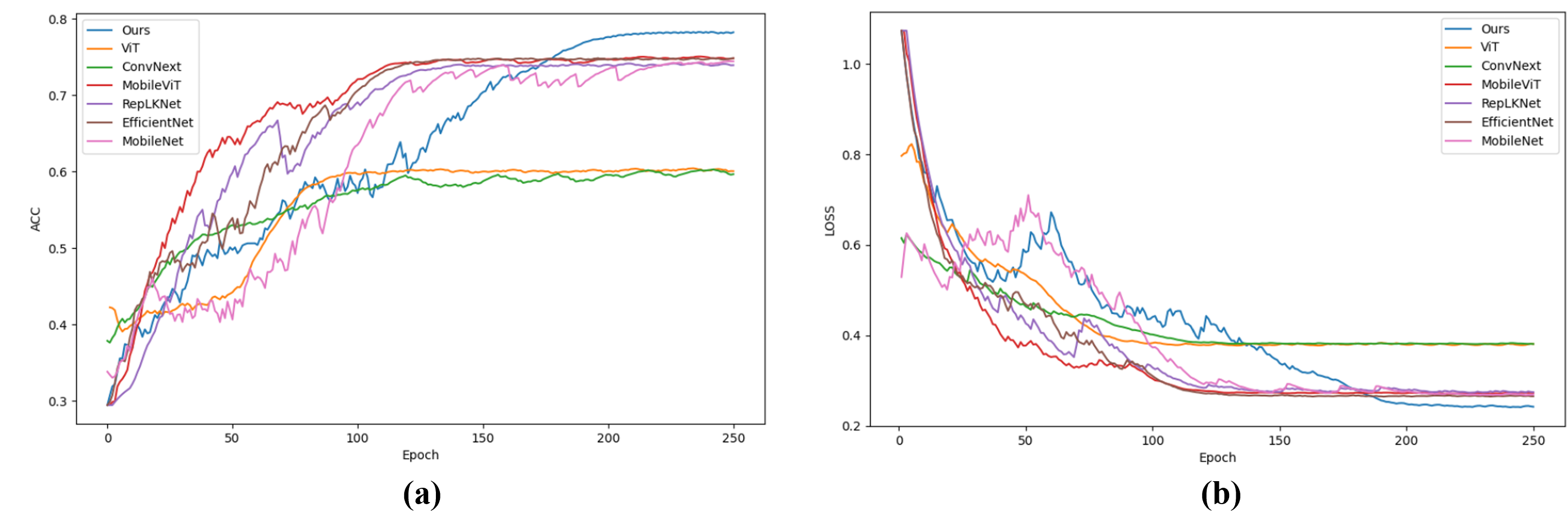


Figure S1. The training results of all models: (a) Accuracy curve; (b) Loss curve.

Note: The basic information of compared models can be found below.

ConvNext is a convolutional neural network (CNN) developed by Facebook AI Research. The main contribution of ConvNext is to upgrade a traditional CNN (e.g., ResNet) by incorporating successful design principles from Vision Transformer (ViT). The initial convolution layer is replaced by a large, non-overlapping convolution layer with a stride of 4, which mimics the way ViTs divide an image into patches. Also, ConvNext utilizes inverted bottlenecks by expanding the feature map with a 1×1 convolution before a depthwise convolution is applied. Furthermore, the standard 3×3 convolution operators are replaced by larger kernels (e.g., 7×7) to increase the receptive field. Besides, ConvNext adopts the layer normalization, rather than batch normalization.

RepLKNet is also a popular CNN and takes advantage of ViTs. It introduces very large convolution kernels (e.g., 31×31) to capture a wider range of contextual information. To make large kernels computationally feasible, RepLKNet adopts a structural reparameterization, which includes a large depth-wise convolution and smaller auxiliary kernels. This helps to reduce latency and make RepLKNet more efficient and practical for real-world applications.

MobileNet is a lightweight and efficient CNN designed by Google. The core innovation of MobileNet is the use of depthwise separable convolutions by breaking down a standard convolution into two much more manageable steps, namely depthwise convolution and pointwise convolution. Decoupling the spatial filtering from the channel combination significantly reduces the number of parameters and computations compared to traditional CNNs. In the following version of MobileNet, inverted residual blocks and linear bottlenecks are introduced in MobileNetv2, while neural architecture search and new activation functions are deployed in MobileNetv3.

EfficientNet is also a lightweight CNN, which balances the prediction accuracy and computational efficiency. Its core innovation is a compound scaling method that systematically scales three dimensions of the network, namely width, depth, and resolution, in a balanced way. It is noted that all these scaling dimensions should be increased in a coordinated manner in order to maximize performance. In this study, we used the baseline model, i.e., EfficientNet-B0.

ViT is a deep learning model that applies the Transformer architecture. ViT represents a significant shift from the traditional CNNs by treating images more like sentences. Given an image, ViT first divides it into small, fixed-size patches (e.g., 16×16). Each patch is then flattened into a vector and linearly projected into a sequence of embeddings. Each patch embedding is also assigned with a learnable positional encoding to enable ViT to understand the spatial information of patches. Then, the patches are fed to a standard Transformer encoder. The core of this encoder is the multi-head self-attention mechanism, which allows to weigh the importance of each patch in relation to all other patches, resulting in capturing long-range dependencies across the entire image. At last, the classification head (typically a simple multi-layer perceptron) makes the decision.

MobileViT is a hybrid neural network architecture that combines ViT and MobileNet. It integrates ViT’s ability to capture global context with the efficiency of CNNs, while maintaining a low computational loads. In terms of local feature extraction, MobileViT starts with a standard convolution layer that processes a small, local region of the image. This helps capture low-level and fine-grained details. After obtaining both local and global features, another convolution layer is used to integrate these two types of information and produce a richer representation.


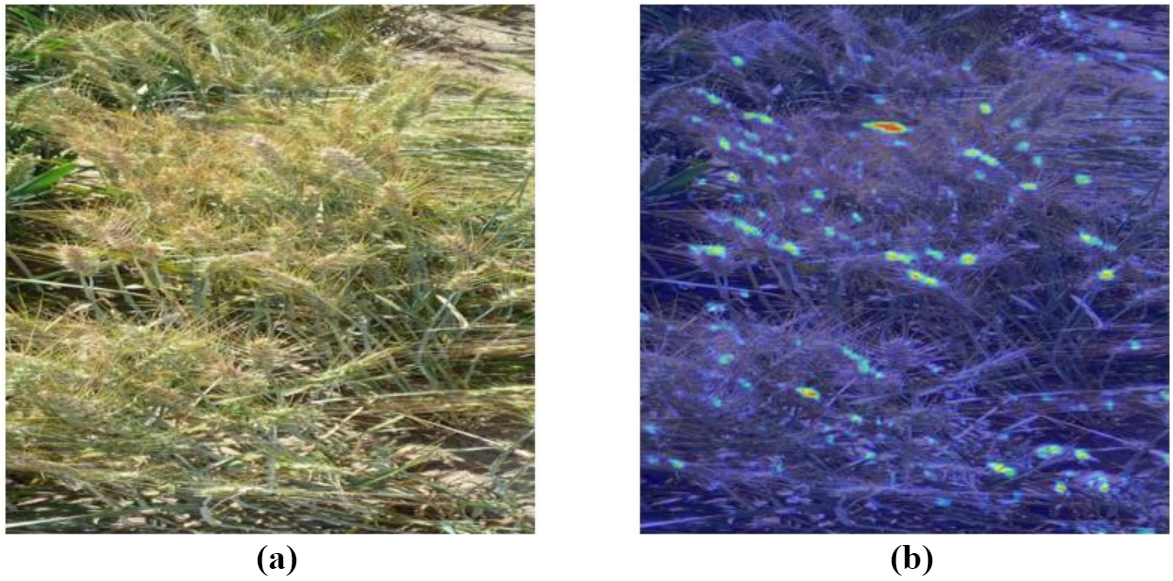


**Figure S2*.*** Failed predictions of FHBNet under the interference of awns and weeds: (a) Original image, (b) Heatmap of FHBNet.
